# Prevalence and Factors Associated with Lifetime HIV Testing Among Thai General Population Adults: Finding from the 6th Thai National Health Examination Survey

**DOI:** 10.3390/healthcare14142230

**Published:** 2026-07-22

**Authors:** Sirinan Prakot, Wichai Aekplakorn, Roengrudee Patanavanich, Masatha Thongpan, Suchanart Inwanna, Donruedee Kamkhoad, Ratchanok Phonyiam, Sukanya Kankaew, Siriphorn Na nakorn, Arissara Sawatpanich, Jittrarath Phothikul

**Affiliations:** 1 Ramathibodi School of Nursing, Faculty of Medicine Ramathibodi Hospital, Mahidol University, Bangkok 10400, Thailand; sirinan.pra@mahidol.ac.th (S.P.); suchanart.inn@mahidol.ac.th (S.I.); donruedee.kak@mahidol.ac.th (D.K.); ratchanok.pha@mahidol.ac.th (R.P.); sukanya.kaw@mahidol.ac.th (S.K.); siriphorn.nan@mahidol.ac.th (S.N.n.); arissara.saa@mahidol.ac.th (A.S.); 2 Department of Community Medicine, Faculty of Medicine Ramathibodi Hospital, Mahidol University, Bangkok 10400, Thailand; wichai.aek@mahidol.ac.th (W.A.); roengrudee.pat@mahidol.ac.th (R.P.); 3 Department of Psychiatry, Faculty of Medicine Ramathibodi Hospital, Mahidol University, Bangkok 10400, Thailand; masatha.tho@mahidol.ac.th

**Keywords:** lifetime HIV testing, Thai adults, general population, National Health Examination Survey, NHES-6, population-based study, Thailand

## Abstract

**Background/Objectives**: HIV testing is essential for early HIV diagnosis, timely treatment initiation, and HIV prevention. Despite Thailand’s long-standing HIV prevention programs and universal access to HIV testing services, nationally representative evidence on lifetime HIV testing among Thai adults remains limited. This study aimed to estimate the prevalence of lifetime HIV testing and identify factors associated with lifetime HIV testing among Thai adults. **Methods:** This study was a secondary analysis of data from the 6th Thai National Health Examination Survey. A total of 11,843 Thai adults aged 20–59 years were included. Descriptive statistics were used to estimate the prevalence of lifetime HIV testing. Survey-weighted logistic regression analyses were performed to examine factors associated with lifetime HIV testing. **Results**: The overall prevalence of lifetime HIV testing was 41.9%, with a prevalence of 42.4% among males, and 41.4% among females. Individuals aged 30–39 years (AOR = 1.64, 95% CI: 1.43–1.87) and 40–49 years (AOR = 1.67, 95% CI: 1.41–1.99), those with secondary education (AOR = 1.64, 95% CI: 1.43–1.87) or certificate/higher education (AOR = 1.74, 95% CI: 1.48–2.05), those currently living with a partner (AOR = 1.60, 95% CI: 1.43–1.79), participants with higher monthly income (10,000–24,999 THB: AOR = 1.13, 95% CI: 1.01–1.27; ≥25,000 THB: AOR = 1.39, 95% CI: 1.13–1.70), residents in the Northern region (AOR = 1.74, 95% CI: 1.45–2.09), and those who had a relative or acquaintance living with HIV/AIDS (AOR = 1.46, 95% CI: 1.34–1.59) were more likely to have lifetime HIV testing. In contrast, Muslim participants (AOR = 0.72, 95% CI: 0.57–0.90), residents of rural areas (AOR = 0.88, 95% CI: 0.79–0.97), residents in the Central (AOR = 0.82, 95% CI: 0.69–0.98) and Southern regions (AOR = 0.83, 95% CI: 0.72–0.94), and those with higher HIV-related stigma (AOR = 0.91, 95% CI: 0.84–0.99) were less likely to have lifetime HIV testing. **Conclusions**: Lifetime HIV testing among Thai adults remained suboptimal, with geographic and sociodemographic disparities.

## 1. Introduction

HIV testing is a critical entry point for ending AIDS. The test enables early diagnosis, timely initiation of antiretroviral therapy, and sustained viral suppression, which improves clinical outcomes and reduces onward transmission [1]. All individuals aged 13–64 are recommended to receive at least one HIV testing in their lifetime, with more frequent testing advised for key populations (e.g., men who have sex with men, transgender women, and sex workers) [2,3]. Despite these recommendations, substantial gaps in testing coverage persist worldwide. The Joint United Nations Programme on HIV/AIDS (UNAIDS) estimates that 40.9 million people worldwide were living with HIV in 2025, of whom approximately 12.0% were unaware of their HIV status [4]. In the United States, as of 2016–2017, approximately 39.0% of the general adult population reported having been being tested for HIV [5]. Thailand has made substantial progress in expanding access to HIV testing and treatment services. All Thai citizens are provided free HIV testing twice a year at public health facilities, community health centers, and specialized clinics nationwide [6]. Despite these efforts, HIV testing coverage was estimated at 53.0% among men who have sex with men, 57.0% among female sex workers, and 38.0% among people who inject drugs [7]. Among the general adult population, although national estimates of HIV testing are rarely reported, a survey study in one province of Thailand found 45.0% (962/2138) of respondents reported having been tested for HIV [8].

Previous studies across diverse settings have shown that HIV testing is associated with multiple factors. Sociodemographic characteristics, including age, sex, marital status, education, income, and employment, are the most reported determinants, with higher testing uptake often observed among women, married individuals, those with higher education, and those with greater socioeconomic resources [9,10,11]. Testing behavior is also associated with perceived risk of HIV infection. Individuals reporting higher-risk behaviors, such as condomless sex, multiple sexual partners, and transactional sex, are more likely to have undergone HIV testing [12]. HIV-related stigma and knowing someone living with HIV have also been identified as important factors influencing testing, with stigma often reducing uptake, while familiarity with people living with HIV is associated with a higher likelihood of testing [8,13,14]. Substance use, particularly alcohol consumption, has also been associated with lower HIV testing uptake in some settings [15,16]. Mental health conditions, including depression and other psychiatric disorders, are associated with HIV testing, with evidence suggesting that those affected have higher rates of HIV testing [17,18,19]. Furthermore, structural factors such as geographic location play an important role, as individuals living in rural or remote areas with limited healthcare infrastructure and transportation barriers are less likely to access HIV testing services [11,20].

In Thailand, existing research regarding HIV testing has largely focused on key populations (e.g., men who have sex with men, transgender women, and sex workers) who are disproportionately affected by HIV and prioritized in national prevention programs [6]. However, nationally representative data on HIV testing behaviors among the general adult population remains limited. This represents an important knowledge gap because general population adults may differ from key populations in HIV risk profiles, perceived need for testing, exposure to targeted interventions, and access to specialized HIV services [21]. As a result, the determinants of HIV testing behavior are likely to differ between these two groups. Without general-population data, it is difficult to identify demographic, geographic, and psychosocial groups that may still be missed by routine testing services. Therefore, this study aimed to estimate the prevalence of HIV testing among Thai general population of adults and to examine factors associated with lifetime HIV testing using data from the 6th Thai National Health Examination Survey (NHES-6).

## 2. Materials and Methods

### 2.1. Study Design, Settings, and Population

This study is a secondary data analysis of existing data from the NHES-6, a nationally representative cross-sectional survey conducted in Thailand during 2019–2020. Comprehensive data on demographic characteristics, health conditions, health behaviors, physical examinations, and laboratory tests were collected from participants of all ages across all regions of Thailand through face-to-face interviews conducted by trained interviewers. A multistage, stratified sampling design was used in NHES-6. Specifically, five provinces were randomly selected from each of Thailand’s four geographic regions. Within each selected province, three to five districts were chosen based on population size, followed by the random selection of villages in rural areas and enumeration areas in urban settings. In the final stage, individuals were selected by age group (1–14 years, 15–59 years, and 60 years and older) and sex using a systematic sampling method, resulting in a total of 32,400 participants [22]. For this study, the analysis was restricted to Thai adults aged 20–59 years (*n* = 11,896), the group for whom HIV testing data were collected. Participants with missing data (*n* = 53) on the main outcome (i.e., lifetime HIV testing) were excluded from the final analytic sample, resulting in a final analytic sample of 11,843 respondents.

### 2.2. Main Outcome

HIV testing was the primary outcome of this study. Lifetime HIV testing was defined as having been tested for HIV. It was assessed based on participants’ self-reported history of lifetime HIV testing and categorized as having been tested versus never tested. Participants who reported having been tested were further asked, “When was your most recent HIV test?” Response options were (a) less than 1 year, (b) 1–2 years, or (c) more than 2 years before the survey. They were also asked, “What was the reason for your most recent HIV test?” Response options were (a) engaging in risky behavior for HIV/AIDS, (b) annual health check-up, (c) job, school, or insurance requirement, and (d) other reasons. Participants who selected “other reasons” were not asked to specify their reasons further; therefore, no additional subcategories or open-text responses were available in the dataset.

### 2.3. Covariates

Covariates were selected based on theoretical considerations, prior literature, and the availability of relevant variables in the NHES-6 dataset. Given that HIV testing is influenced by multiple interrelated factors, the socio-ecological model (SEM) was applied to guide covariate selection, organization, and interpretation of study findings [23]. Using the SEM, HIV testing was conceptualized as a behavior shaped by individual, interpersonal, and broader contextual influences. Sociodemographic factors (i.e., sex, age group, marital status, religion, education, occupation, and monthly income) and behavioral and psychosocial factors (i.e., binge drinking, depressive symptoms, HIV-related stigma) were included as individual-level determinants of HIV testing decisions. Familiarity with individuals living with HIV was included as an interpersonal-level factor reflecting social exposure to HIV. Geographic factors (i.e., region and residential area) were included as contextual indicators of potential differences in healthcare access, service availability, and HIV testing infrastructure.

Binge drinking behavior was assessed with the question: “During the past 30 days, have you consumed six or more standard drinks on one occasion, such as more than four cans or two large bottles of beer, three or more shots of spirits, or six glasses of wine?” Response options were “yes” and “no.” Participants who responded “yes” were classified as having engaged in binge drinking behavior during the past 30 days.

Depressive symptoms were assessed using the Thai version of Patient Health Questionnaire-9 (PHQ-9), a validated nine-item screening instrument designed to measure the depressive symptoms over the past two weeks. Each item is scored on a four-point Likert scale ranging from 0 (“not at all”) to 3 (“nearly every day”), yielding a total score ranging from 0 to 27. A cut-off score of 10 or greater has been recommended for identifying clinically significant depressive symptoms and screening for major depression [24]. In this study, PHQ-9 scores were categorized into two groups: no to mild depressive symptoms (total score = 0–9) and moderate to severe depressive symptoms (total score = 10–27). The Thai version of the PHQ-9 demonstrated acceptable internal consistency, with a Cronbach’s alpha of 0.79 [25].

HIV-related stigma was assessed using six standardized items adapted from the Global Stigma and Discrimination Indicators Working Group (GSDIWG) draft report. The items evaluated participants’ agreement with statements reflecting anticipated stigma, perceived stigma, fear of HIV infection, social judgment, experienced stigma, and discrimination [26]. Details of the measurement have been published elsewhere [27]. The total HIV-related stigma was calculated by summing affirmative responses across the six items. Total scores ranged from 0 to 6, with higher scores indicating greater levels of stigmatizing attitudes toward people living with HIV/AIDS. The total score was dichotomized as lower versus higher stigma. Scores below the mean were classified as lower HIV-related stigma, whereas scores at or above the mean were classified as higher HIV-related stigma. The six-item HIV-related stigma measure has demonstrated acceptable internal consistency in a prior Thai population-based study, with a Cronbach’s alpha of 0.72 [28]. In the current sample, the measure had a Cronbach’s alpha of 0.62.

### 2.4. Statistical Analysis

All analyses were conducted in Stata (version 16) and accounted for the complex multistage sampling design of NHES-6 by using the *svy* prefix command. Descriptive statistics were calculated and are presented as frequencies and percentages for categorical variables and as means with standard deviations (SD) for continuous variables. Differences in weighted prevalence of lifetime HIV testing according to participant characteristics were assessed using survey-adjusted chi-square tests.

Survey-weighted logistic regression was used to identify factors associated with lifetime HIV testing. Candidate variables for the multivariable model were selected based on prior literature, theoretical relevance to HIV testing, and evidence of association in univariable analyses. The *p* < 0.20 criterion was used as a liberal screening guide to avoid prematurely excluding potentially important predictors. Missing data were assessed for all covariates and handled using multiple imputation by chained equations under the missing-at-random assumption. Twenty imputed datasets were generated. The imputed variables included education level, marital status, religion, occupation, monthly income, binge drinking in the past 30 days, depressive symptoms, and HIV-related stigma. The chained-equation imputation models included sex, age group, region, and residential area as predictors, together with the other variables included in the imputation procedure. Survey weights were not incorporated into the imputation process. The final multivariable model was estimated using multiply imputed survey-weighted logistic regression. Convergence was assessed by examining trace plots of the means and standard deviations of imputed variables across iterations. The trace plots showed stable fluctuations without systematic upward or downward trends, suggesting adequate convergence. Multicollinearity was assessed using variance inflation factors (VIFs). No evidence of serious multicollinearity was observed, as all VIF values were below 5. Complete-case analysis was conducted as a sensitivity analysis and yielded findings substantively similar to the multiple imputation analysis. Results are reported as odds ratios (ORs) and adjusted odds ratios (AORs) with 95% confidence intervals (CIs). All statistical tests were two-sided, and *p* < 0.05 was considered statistically significant.

### 2.5. Ethical Considerations

The original NHES-6 survey received ethical approval, and informed consent was obtained from participants at the time of data collection. This study used de-identified secondary data of the NHES-6 and received independent ethical approval from the Institutional Review Boards of Faculty of Medicine Ramathibodi Hospital, Mahidol University on 24 July 2025 (No. MURA2025/604).

## 3. Results

### 3.1. Participant Characteristics

A total of 11,843 adults aged 20–59 years were included in the analysis. Participants were recruited from all regions of Thailand. Most participants were female (51.1%), with a mean age of 42.08 years (SD = 11.54). Approximately 40.7% had completed secondary education, and 94.6% identified as Buddhist. Over half (61.3%) were engaged in manual or informal employment (e.g., agriculture, labor, service, or self-employment) and 46.7% reported a monthly income of equal or less than 9999 THB (approximately 300 USD). Detailed participant characteristics are presented in Table 1.

### 3.2. Lifetime HIV Testing

As shown in Table 2, the prevalence of lifetime HIV testing among Thai adults was 41.9%. Among respondents who had been tested for HIV, 67.5% reported that their most recent HIV test had occurred more than two years before the survey. The most commonly reported reason for the most recent HIV test among those who had been tested was “other reasons” (47.1%), followed by “annual health check-ups” (37.8%).

The comparison of participant characteristics by lifetime HIV testing status are presented in Table 1. HIV testing prevalence was higher among individuals aged 30–39 years and 40–49 years than among those aged 20–29 years and 50–59 years (50.0% and 48.9% vs. 34.8% and 35.4%, respectively; *p* < 0.001). Higher testing prevalence was also observed among participants with secondary or higher education (45.9–47.9% vs. 34.5%, *p* < 0.001), those currently living with a partner (45.6% vs. 34.2%, *p* < 0.001), and those with monthly income ≥ 25,000 THB compared with ≤9999 THB (53.5% vs. 36.0%, *p* < 0.001).

By region, participants residing in the North had the highest HIV testing prevalence (55.2%), whereas lower prevalence was observed in the Central and South regions (both 36.1%; *p* < 0.001). HIV testing prevalence also differed significantly according to religion, depressive symptoms, HIV-related stigma, and having a relative or acquaintance living with HIV/AIDS (all *p* < 0.001), as well as occupation (*p* = 0.014) and binge drinking status (*p* = 0.009). Participants with moderate to severe depressive symptoms had lower HIV testing prevalence than those with no to mild depressive symptoms (29.5% vs. 42.0%, *p* = 0.007), whereas participants who had a relative or acquaintance living with HIV/AIDS had higher testing prevalence than those who did not (51.1% vs. 39.1%, *p* < 0.001). No significant differences were observed according to sex or residential area.

### 3.3. Factors Associated with Lifetime HIV Testing

Table 3 presents the results of the survey-weighted logistic regression analyses. In univariable analyses, most factors were significantly associated with lifetime HIV testing (*p* < 0.05), except for sex (*p* = 0.286) and residential area (*p* = 0.412). However, both variables were retained in the multivariable model because of their theoretical importance and potential role as confounding factors.

In the multivariable model, adults aged 30–39 years (AOR = 1.64, 95% CI: 1.43–1.87) and 40–49 years (AOR = 1.67, 95% CI: 1.41–1.99) had higher odds of lifetime HIV testing than those aged 20–29 years, while no significant difference was observed for those aged 50–59 years (AOR = 1.08, 95% CI: 0.92–1.26). Higher education (secondary: AOR = 1.64, 95% CI: 1.43–1.87; certificate or higher: AOR = 1.74, 95% CI: 1.48–2.05), living with a partner (AOR = 1.60, 95% CI: 1.43–1.79), residence in the North (AOR = 1.74, 95% CI: 1.45–2.09), higher income (10,000–24,999 THB: AOR = 1.13, 95% CI: 1.01–1.27; ≥25,000 THB: AOR = 1.39, 95% CI: 1.13–1.70), and having a relative or acquaintance living with HIV/AIDS (AOR = 1.46, 95% CI: 1.34–1.59) were associated with higher odds of testing. Lower odds were observed among those residing in the Central (AOR = 0.82, 95% CI: 0.69–0.98) and South (AOR = 0.83, 95% CI: 0.72–0.94) regions, those living in rural areas (AOR = 0.88, 95% CI: 0.79–0.97), those identifying as Muslim (AOR = 0.72, 95% CI: 0.57–0.90), and those with higher HIV-related stigma (AOR = 0.91, 95% CI: 0.84–0.99). Sex, region of residence in the Northeast, occupation, binge drinking, depressive symptoms, Christianity, and uncertainty regarding whether a relative or acquaintance was living with HIV/AIDS were not statistically significant in the multivariable model.

## 4. Discussion

This study estimated the prevalence of lifetime HIV testing and examined its associated factors among Thai general adults using nationally representative NHES-6 data. Our findings showed that only 41.9% of Thai general population adults have undergone HIV testing, suggesting that more than half of the population had never been tested for HIV. The UNAIDS 95–95–95 target specifies that at least 95% of people living with HIV should know their HIV status [29]. However, this indicator applies to people living with HIV. No global recommendation specifies a target percentage of HIV testing for the general population. HIV testing goals should therefore be risk-based and context-specific [1]. In Thailand, where HIV testing strategies include both population-level access and targeted services for groups with higher HIV vulnerability, lifetime testing prevalence provides important evidence on population-level engagement with HIV testing. Higher lifetime HIV testing among general population adults can facilitate earlier diagnosis, timely linkage to care, and identification of undiagnosed infections among individuals who may not perceive themselves to be at risk. Therefore, the finding that fewer than half of Thai adults have undergone HIV testing suggests substantial gaps in HIV testing exposure among Thai adults and highlights the need to strengthen population-accessible and context-specific HIV testing strategies in Thailand.

The prevalence of HIV testing in the present NHES-6 study was lower than estimates reported in earlier studies, including 47.9% in the 2006 National Sexual Behavior Survey among adults aged 18–59 years [30] and 45.0% in a 2012 population-based study conducted in Nonthaburi Province among adults aged 15–59 years [8]. Although direct comparisons across these studies should be interpreted cautiously due to differences in study design and study populations, these findings may suggest limited progress in improving HIV testing uptake among Thai adults over the past decade. Future longitudinal and repeated national surveys are needed to confirm the actual trend in HIV testing uptake among Thai adults.

Compared with international studies, the prevalence of lifetime HIV testing observed in our study was slightly lower than estimates reported in the United States, where approximately 45.9% of adults have undergone HIV testing [31]. Within Southeast Asia, the observed prevalence was comparable to that reported in Cambodia (42.1%) and higher than estimates from Myanmar (19.5%), the Philippines (4.6%), and Timor-Leste (3.7%) [11]. However, these cross-country comparisons should be interpreted with caution because the cited studies differed in survey design, study population, age range, timing of data collection, and measurement approaches. Beyond these methodological differences, variations in HIV epidemiology, healthcare infrastructure, and national HIV testing policies across countries may also contribute to differences in HIV testing prevalence. In the United States, routine HIV screening has long been recommended in healthcare settings regardless of perceived HIV risk [32]. Similarly, Thailand and Cambodia have implemented extensive HIV prevention and testing programs, including integration of HIV services into routine healthcare and expansion of community-based HIV services [33,34]. In summary, these approaches may have contributed to relatively higher HIV testing uptake compared with neighboring countries in Southeast Asia.

Geographic disparities in HIV testing were also observed in our findings. Rural residents were less likely to report having been tested for HIV than urban residents. Similar disparities have been reported in other studies [11,20,35], suggesting that individuals living in urban areas or areas with better healthcare infrastructure may have greater access to HIV testing services and related health resources. In addition, individuals residing in the Northern region demonstrated the highest HIV testing prevalence (55.2%), whereas a lower prevalence was observed in the Central and Southern regions (both 36.1%). Although Bangkok is the capital city and has the highest concentration of healthcare facilities and HIV testing services in Thailand, individuals residing in the North had higher odds of HIV testing than those residing in Bangkok. One possible explanation is the historical impact of the HIV epidemic in Northern Thailand, which prompted strong community engagement and targeted interventions during the early epidemic period [36]. These factors may be associated with greater awareness and familiarity with HIV services, potentially increasing higher testing uptake. However, since this study did not directly measure historical community engagement or regional program intensity, future studies are needed to explore these regional disparities in greater detail. Nevertheless, this study’s findings highlight the need for effective, region-specific strategies to enhance equitable access to HIV testing nationwide, especially in rural areas and regions with lower testing prevalence, such as the Central and Southern regions.

Age was another important factor associated with lifetime HIV testing. HIV testing prevalence was highest among adults aged 30–39 years (50.0%) and 40–49 years (48.9%), whereas considerably lower prevalence was observed among younger adults aged 20–29 years (34.8%) and older adults aged 50–59 years (35.4%). This age-related pattern may reflect differences in opportunities for HIV testing through routine healthcare contact. In this study, annual health check-ups were the second most common reason for HIV testing among participants who had been tested (37.8%), followed by job-, school-, or insurance-related requirements (8.9%). These findings suggest that HIV testing among Thai adults may occur through routine or administrative health encounters, which may provide greater testing opportunities for middle-aged adults. Similarly, lower testing prevalence among younger and older adults may reflect fewer routine testing opportunities. Similar age-related patterns have been reported in previous studies, in which HIV testing uptake tends to increase with healthcare utilization [10,37]. However, it is important to note that healthcare utilization patterns and individual motivations for testing were not measured in this study. In addition, because HIV testing behavior is complex and may be influenced by other factors, such as perceived risk and sexual behaviors, which were not measured in NHES-6, these age-related differences should be interpreted cautiously. Future research should include these indicators, along with more detailed measures of testing motivation, to better understand pathways underlying HIV testing uptake across age groups. Nevertheless, the observed lower testing prevalence among younger and older adults suggests that these age groups may remain insufficiently reached by current HIV testing strategies in Thailand.

Similar to the prior literature [38], higher educational attainment and income were associated with higher odds of lifetime HIV testing among Thai general population adults. HIV testing prevalence increased from 34.5% among individuals with primary education or lower to 47.9% among those with certificate-level education or higher. Similarly, testing prevalence increased from 36.0% among individuals earning ≤ 9999 THB per month to 53.5% among those earning ≥ 25,000 THB. These associations may reflect differences in health literacy, awareness of HIV prevention, and access to healthcare resources. Individuals with higher educational attainment may have greater health literacy, enabling them to better understand HIV transmission, recognize personal risk, and engage in preventive behaviors such as HIV testing [39]. Higher income levels may also reduce financial and structural barriers to healthcare access, including transportation costs, opportunity costs, and access to higher-quality health services [40]. However, these interpretations should be made cautiously because HIV risk-related variables, including sexual behavior, perceived HIV risk, condom use, history of sexually transmitted infections, sexual orientation, and HIV knowledge, were not available in the NHES-6 dataset. The absence of these variables may have resulted in residual confounding and limits interpretation of the observed socioeconomic associations. Together, these findings highlight the persistence of socioeconomic inequalities in HIV testing uptake in Thailand.

Thai adults living with a partner had 60% higher odds of testing than those who did not. Similar findings have been reported in Southeast Asia [11] and sub-Saharan Africa [41], where marital status has been associated with a higher testing uptake. One possible explanation is that adults living with a partner may have more opportunities for healthcare contact, such as reproductive care or routine check-ups, and may have greater awareness of HIV risk within relationships. In addition, participants who had a relative or acquaintance living with HIV/AIDS had a higher prevalence of lifetime HIV testing than those who did not and the overall study population (51.1% vs. 39.1% and 41.9%, respectively). That is, having a relative or acquaintance living with HIV/AIDS was associated with 46% higher odds of lifetime HIV testing. This finding is consistent with previous studies conducted in Thailand [8] and Italy [14]. Personal exposure to HIV may be associated with greater awareness or perceived susceptibility, which may in turn be related to testing uptake [42]. However, given the cross-sectional design, the temporal direction of this association cannot be established.

Regarding religion, Thai adults identifying as Muslim had 28% lower odds of having been tested for HIV compared with those identifying as Buddhist. One possible explanation is the presence of stigma associated with HIV within Muslim contexts. A previous study suggests that, in some Muslim communities, HIV may be interpreted as a consequence of behaviors perceived to violate religious or moral norms, such as extramarital sexual activity or substance use [43]. However, the present study did not directly measure religiosity, cultural norms, HIV-related beliefs, or group-specific barriers to HIV testing. Future qualitative and mixed-methods studies are needed to better understand HIV testing decisions among Muslim populations in Thailand.

Higher HIV-related stigma was significantly associated with lower odds of lifetime HIV testing in the adjusted model. This finding is consistent with prior studies showing that HIV-related stigma can act as a barrier to HIV testing by discouraging individuals from seeking testing services [13]. However, it is important to note that the stigma measure showed modest internal consistency in the current sample (Cronbach’s alpha = 0.62), which may have affected the precision of the estimate. In addition, the NHES-6 dataset measured general HIV-related stigmatizing attitudes toward people living with HIV, as described elsewhere [27]. However, it did not capture testing-specific stigma, such as concerns about confidentiality, fear of being seen at HIV testing sites, anticipated negative reactions following a positive result, or fear of disclosure. Therefore, future studies using more comprehensive and testing-specific stigma measures are needed to better understand how different dimensions of HIV-related stigma influence HIV testing behavior among Thai adults.

Notably, sex, occupation, binge drinking, and depressive symptoms were not significantly associated with lifetime HIV testing after controlling for other factors. However, these non-significant findings should be interpreted with caution. Prior studies have reported associations between HIV testing and these factors [9,15,19]. In the present study, some categories were small, particularly respondents with moderate-to-severe depressive symptoms (*n* = 99) and Christian respondents (*n* = 100), which may have resulted in limited statistical power and reduced the stability of the estimates. In addition, residual confounding may have influenced the observed associations because several HIV risk-related variables were not available in the NHES-6 dataset. Measurement limitations and residual confounding may therefore have contributed to the non-significant findings. Future studies with larger subgroup samples and more detailed measures of HIV risk and prevention-related factors are needed to clarify the roles of these non-significant variables and HIV risk profiles in HIV testing behavior among Thai adults.

### 4.1. Implications for Practice and Policy

The findings of this study have important implications for HIV prevention and testing strategies in Thailand. Given the relatively low prevalence of lifetime HIV testing among Thai general population adults, strengthening HIV testing uptake in the general population remains necessary. Based on the observed patterns of lower testing uptake, future interventions may consider focusing on younger adults aged 20–29 years, older adults aged 50–59 years, individuals with lower educational attainment and income, and those residing in the Central and Southern regions. Possible strategies may include expanding access to convenient, confidential, and stigma-sensitive testing options, such as HIV self-testing and community-based testing. Integrating HIV testing into routine healthcare services and annual health check-ups may also increase opportunities for early diagnosis. In addition, public health campaigns aimed at improving HIV awareness and reducing HIV-related stigma may help increase testing uptake. Together, these strategies may facilitate earlier HIV diagnosis, improve timely linkage to care, and support Thailand’s progress toward ending AIDS by 2030.

### 4.2. Strengths and Limitations

This study has notable strengths and limitations. An important strength was the use of nationally representative data from the Thai NHES-6, allowing the findings to be generalizable to the adult population in Thailand. In addition, the standardized data collection procedures, together with the use of survey weighting and multiple imputation, strengthened the validity and reliability of the findings. Therefore, this study provides benchmark population-level data on HIV testing in Thailand, which can serve as a reference for monitoring progress and informing future policies and intervention.

However, some limitations should be considered. First, this secondary analysis relied on existing data, and several important HIV risk-related variables were not available, including sexual behavior, perceived HIV risk, history of sexually transmitted infections, condom use, sexual orientation, and HIV knowledge. The absence of these variables limits interpretation of the associated factors and may have resulted in residual confounding factors. In addition, although the SEM was used to guide covariate selection and interpretation, NHES-6 mainly captured individual-level factors and limited interpersonal and contextual indicators. Organizational, community, and policy-level determinants, such as facility-level HIV testing availability, local prevention programs, community HIV awareness campaigns, and implementation of HIV testing policies, were not available and therefore could not be evaluated. Second, this study used lifetime HIV testing as the primary outcome, which captures whether participants had ever accessed HIV testing but does not reflect recent testing, repeat testing, or current engagement with HIV testing services. Therefore, lifetime testing may overestimate current testing coverage and should not be interpreted as evidence of adequate routine or recent testing. Third, some subgroups were small, particularly respondents with moderate-to-severe depressive symptoms (*n* = 99) and Christian respondents (*n* = 100), which may have resulted in limited statistical power and reduced the stability of the estimates. Therefore, findings for these subgroups should be interpreted with caution. Fourth, all variables were self-reported and may be subject to recall or social desirability bias. Finally, due to the cross-sectional design, causal relationships cannot be established, and the temporal direction between associated factors and HIV testing requires further investigation.

## 5. Conclusions

This study provides nationally representative evidence on lifetime HIV testing among Thai general population adults and identifies key factors associated with testing uptake. Overall, HIV testing remains suboptimal among Thais, with fewer than half of adults reporting having been tested. Higher odds of reporting lifetime HIV testing were observed among adults aged 30–49 years, individuals with higher educational attainment and income, those living with a partner, residents in the Northern region, and those who had a relative or acquaintance living with HIV/AIDS. In contrast, lower uptake was observed among rural residents, individuals in certain regions, those identifying as Muslim, and those reporting higher HIV-related stigma. These findings highlight persistent geographic and sociodemographic disparities in HIV testing across Thailand and underscore the need for more equitable and population-wide HIV testing strategies.

## Figures and Tables

**Table 1:** Participant Characteristics and Prevalence of HIV Testing (*N* = 11,843).

Variable	Overall		Tested			
	n	% *	n	% *	95% CI	*p* ^1^
**Sex**						0.285
Male	4620	48.9	1957	42.4	40.6–44.3	
Female	7223	51.1	2990	41.4	39.3–43.5	
**Age (years)**						<0.001
20–29	2221	18.4	750	34.8	31.6–38.1	
30–39	2718	22.8	1380	50.0	47.9–52.1	
40–49	2849	24.4	1362	48.9	45.6–52.2	
50–59	4055	34.4	1455	35.4	33.9–37.0	
(min–max = 20–59; mean = 42.08; SD = 11.54)						
**Education Level**						<0.001
Primary or lower	4294	38.8	1486	34.5	33.0–36.0	
Secondary	4746	40.7	2178	45.9	43.5–48.3	
Certificate or higher	2748	20.5	1256	47.9	44.4–51.4	
Missing	55	-	27	-	-	
**Marital Status**						<0.001
Not currently living with partner	4153	33.9	1430	34.2	32.1–36.5	
Currently living with partner	7494	66.1	3433	45.6	43.6–47.7	
Missing	196	-	84	-	-	
**Religion**						<0.001
Buddhism	10,928	94.6	4652	42.3	40.7–44.0	
Christianity	100	1.0	49	52.1	42.4–61.7	
Islam	803	4.4	239	30.0	24.3–36.3	
Missing	12	-	7	-	-	
**Occupation**						0.014
Professional/Formal employment	2642	22.0	1192	45.4	42.2–48.6	
Manual/Informal employment	6194	61.3	2529	41.1	38.9–43.3	
Student/Unemployed	2074	16.7	807	38.8	35.5–42.2	
Missing	933	-	419	-	-	
**Monthly Income (THB)**						<0.001
≤9999	4315	46.7	1564	36.0	33.7–38.3	
10,000–24,999	3825	41.7	1667	43.7	41.7–45.7	
≥25,000	1034	11.6	524	53.5	48.9–58.1	
Missing	2669	-	1192	-	-	
**Region**						<0.001
North	2421	18.0	1327	55.2	52.2–58.1	
Central	2721	26.2	936	36.1	33.6–38.8	
Northeast	2795	33.0	1187	41.9	39.7–44.2	
South	2549	14.0	922	36.1	33.4–38.9	
Bangkok	1357	8.8	575	41.2	39.8–42.5	
**Residential Area**						0.411
Urban	6549	35.6	2863	42.6	40.8–44.3	
Rural	5294	64.4	2084	41.5	39.3–43.9	
**Binge Drinking in Past 30 Days**						0.009
No	10,468	86.4	4313	41.4	39.5–43.3	
Yes	1369	13.6	630	45.3	43.0–47.5	
Missing	6	-	4	-	-	
**Depressive Symptom**						0.007
No to mild depression	11,730	99.2	4905	42.0	40.3–43.8	
Moderate to severe depression	99	0.8	35	29.5	22.4–37.7	
Missing	14	-	7	-	-	
(min–max = 0–25; mean = 1.91; median = 1; SD = 2.68)						
**Stigma**						<0.001
<mean	5215	44.2	2370	44.1	42.2–46.1	
≥mean	6623	55.8	2576	40.1	38.2–42.0	
Missing	5	-	1	-	-	
(min–max = 0–6; mean = 2.88; SD = 1.60)						
**Having an HIV/AIDS-infected Relative or** **Acquaintance**						<0.001
Yes	2834	23.8	1440	51.1	48.3–54.0	
No	8846	74.7	3443	39.1	37.6–40.7	
Not sure	163	1.5	64	32.9	24.7–42.4	

* Survey-weighted proportion; ^1^ *p*-values were obtained from survey-adjusted chi-square tests comparing the prevalence of HIV testing across participant characteristics.

**Table 2:** Lifetime HIV testing among Thai adults aged 20–59 years (*N* = 11,843).

Testing Characteristic	n	% *	95% CI
**Lifetime HIV Testing**			
Tested	4947	41.9	40.2–43.6
Never tested	6896	58.1	56.4–59.8
**Time since the most recent HIV test among respondents who had been tested (*n* = 4947)**			
<1 year ago	768	14.8	13.5–16.1
1–2 years ago	949	17.7	16.5–19.0
>2 years ago	3230	67.5	65.7–69.3
**Reason for the most recent HIV test among respondents who had been tested (*n* = 4947)**			
Had a risky behavior	282	6.2	5.2–7.3
Annual health check-up	1840	37.8	36.0–39.7
Job/school/insurance requirement	385	8.9	7.7–10.3
Other reasons	2440	47.1	44.9–49.3

* Survey-weighted proportion; “Other reasons” includes responses that did not fall under the specified categories. No additional subcategories or open-text responses were available in the dataset.

**Table 3:** Survey-weighted univariable and multivariable logistic regression analyses of factors associated with lifetime HIV testing (*N* = 11,843).

Factor	Univariable			Multivariable		
	OR	95% CI	*p*-Value	AOR	95% CI	*p*-Value
**Sex** (ref: male)						
Female	0.96	0.88–1.04	0.286	0.95	0.86–1.05	0.298
**Age** (years; ref: 20–29)						
30–39	1.87	1.63–2.15	<0.001	1.64	1.43–1.87	<0.001
40–49	1.79	1.50–2.14	<0.001	1.67	1.41–1.99	<0.001
50–59	1.03	0.88–1.20	0.699	1.08	0.92–1.26	0.349
**Education** (ref: Primary and lower)						
Secondary	1.61	1.45–1.79	<0.001	1.64	1.43–1.87	<0.001
Certificate or higher	1.74	1.53–1.98	<0.001	1.74	1.48–2.05	<0.001
**Marital status** (ref: Not currently living with partner)						
Currently living with partner	1.59	1.42–1.77	<0.001	1.60	1.43–1.79	<0.001
**Religion** (ref: Buddhism)						
Christianity	1.48	1.02–2.15	0.041	1.15	0.83–1.60	0.372
Islam	0.58	0.44–0.78	0.001	0.72	0.57–0.90	0.008
**Region** (ref: Bangkok)						
North	1.76	1.54–2.01	<0.001	1.74	1.45–2.09	<0.001
Central	0.81	0.71–0.92	0.003	0.82	0.69–0.98	0.030
Northeast	1.03	0.92–1.15	0.561	1.15	0.99–1.33	0.062
South	0.81	0.71–0.92	0.004	0.83	0.72–0.94	0.006
**Residential Area** (Ref: Urban)						
Rural	0.96	0.86–1.07	0.412	0.88	0.79–0.97	0.014
**Occupation** (ref: Professional/Formal employment)						
Manual/Informal employment	0.85	0.72–1.00	0.052	0.95	0.78–1.17	0.629
Student/Unemployed	0.76	0.63–0.92	0.009	1.00	0.82–1.23	0.959
**Income** (THB; ref: ≤9999)						
10,000–24,999	1.28	1.12–1.45	0.002	1.13	1.01–1.27	0.031
≥25,000	1.82	1.48–2.22	<0.001	1.39	1.13–1.70	0.004
**Binge drinking in past 30 days** (ref: No)						
Yes	1.17	1.05–1.32	0.010	1.08	0.97–1.21	0.144
**Current depressive symptom** (ref: No to mild depression)						
Moderate to severe depression	0.58	0.39–0.86	0.009	0.71	0.46–1.11	0.122
**Stigma** (ref: <mean)						
≥mean	0.85	0.78–0.92	<0.001	0.91	0.84–0.99	0.025
**Have a relative/acquaintance living with HIV/AIDS** (ref: No)						
Yes	1.63	1.45–1.82	<0.001	1.46	1.34–1.59	<0.001
Not sure	0.76	0.51–1.15	0.183	0.71	0.46–1.10	0.116

## Data Availability

The data are not publicly available. The dataset was obtained from the NHES Project, Department of Community Medicine, Faculty of Medicine Ramathibodi Hospital, Mahidol University, Thailand, and was used under permission for the present study. Data access requests should be directed to the NHES Project at https://thai-nhes.com and are subject to approval by the data custodian.

## Abbreviations

The following abbreviations are used in this manuscript:

AIDS	Acquired Immunodeficiency Syndrome
AOR	Adjusted odds ratio
CI	Confidence interval
HIV	Human Immunodeficiency Virus
NHES	Thai National Health Examination Survey
NHES-6	The 6th Thai National Health Examination Survey
OR	Odds ratio
PHQ-9	Patient Health Questionnaire-9
SD	Standard deviation
THB	Thai baht
UNAIDS	The Joint United Nations Programme on HIV/AIDS
USD	United States Dollar
